# Variants of *OTOF* and *PJVK* Genes in Chinese Patients with Auditory Neuropathy Spectrum Disorder

**DOI:** 10.1371/journal.pone.0024000

**Published:** 2011-09-15

**Authors:** Wang Jian, Fan Ying-ying, Wang Shu-juan, Liang Peng-Fei, Wang Jin-ling, Qiu Jian-hua

**Affiliations:** 1 Deafness Gene Diagnosis, PLA Otolaryngology-Head and Neck Surgery Center, Xijing Hospital, Fourth Military Medical University, Xi'an, Shannxi, China; 2 Department of Anesthesiology, Stomatological College, Fourth Military Medical University, Xi'an, Shannxi, China; 3 PLA Otolaryngology-Head and Neck Surgery Center, Xijing Hospital, Fourth Military Medical University, Xi'an, Shannxi, China; Hotchkiss Brain Institute, University of Calgary, Canada

## Abstract

**Background:**

Mutations in *OTOF* and *PJVK* genes cause DFNB9 and DFNB59 types of hearing loss, respectively. The patients carrying pathogenic mutations in either of these genes may show the typical phenotype of auditory neuropathy spectrum disorder (ANSD). The aim of the present study was to identify *OTOF* and *PJVK* mutations in sporadic ANSD patients.

**Methods and Findings:**

A total of 76 unrelated Chinese non-syndromic ANSD patients were sequenced on the gene *OTOF* and *PJVK* exon by exon. Variants were valued in 105 controls with normal hearing to verify the carrying rate. We identified one pathogenic mutation (c.1194T>A) and three novel, possibly pathogenic, variants (c.3570+2T>C, c.4023+1 G>A, and c.1102G>A) in the *OTOF* gene, and one novel, possibly pathogenic, variant (c.548G>A) in *PJVK*. Moreover, we found three novel missense mutations within the exons of *OTOF*.

**Conclusions:**

As we identified 4 and 1 possible pathogenic variants of the OTOF gene and the PJVK gene, respectively, we believe that screening in these genes are important in sporadic ANSD patients. The pathogenicity of these novel mutations needs further study because of their single heterozygous nature. Knowledge on the mutation spectra of these genes in Chinese would be beneficial in understanding the genetic character of this worldwide disease.

## Introduction

Auditory neuropathy (AN), also known as auditory dys-synchrony [1], is a special type of sensorineural hearing disorder. This disorder defined and nominated by Starr in 1996, based on the characters in auditory tests [2]. The terminology of auditory neuropathy spectrum disorder (ANSD) was recommend to name the disease in 2008 due to its heterogeneous and multifaceted characteristics [3]. In patients with ANSD, otoacoustic emissions are normal or partly normal, which reflect the preservation of function in outer hair cells (OHCs). However, their auditory brainstem responses (ABRs) are profoundly abnormal or absent, indicating that the disorder lies either in the inner hair cells (IHCs), in the intervening synapse, or in the auditory nerve [2], [4], [5]. The pure-tone audiometric results in ANSD patients can vary greatly from normal hearing to severe hearing loss, but the thresholds are elevated higher in the low-frequency region in most of the patients. The major complaint of ANSD patients is their difficulty in understanding words, especially of loud voices or in noisy environments [5].

The prevalence of ANSD in sensorineural hearing loss (SNHL) can range from 2.4% to 15%, which may be due to the varied criteria by different researchers [6]. Rance et al. [7] described a 23% prevalence of ANSD within the at-risk neonates. Our former study showed that 1.37% of patients with SNHL are diagnosed with ANSD [8]. The etiologies of ANSD can be classified into three main groups, namely, genetic, infectious, and neonatal/prenatal risk factors [3], [9], [10]. Although histories of infections or risk factors are responsible for some ANSD patients, defined etiology are rare in majority of these patients, especially those with hearing loss onset in the second or the third decade [8]. Congenital ANSD, syndromic or non-syndromic, are closely related with genetic abnormality [3]. Mutations in either the *OTOF* (Gene ID: 9381, MIM*603681) or *PJVK* gene (Gene ID: 494513, MIM*610219) can cause congenital recessive ANSD [11], [12]. Additionally, mutations in *GJB2* (Gene ID: 2706, MIM*121011) and mitochondrial *12S rRNA* (Gene ID: 4549, MIM*561000) have also been detected as the cause of ANSD in some patients [13], [14].

The *OTOF* gene is responsible for DFNB9 (MIM#601071) [15] and the non-syndromic recessive ANSD [12]. *OTOF* gene mutations have been inferred to be responsible for 2%–3% non-syndromic hearing losses (NSHL) in some ethnic groups, and most of these patients meet the diagnostic criteria for ANSD [16], [17]. Otoferlin, encoded by *OTOF*, is critical for exocytosis at the auditory ribbon synapse in a calcium-dependent manner [18], [19]. To date, more than 60 pathogenic variants of the *OTOF* gene have been reported in familial or sporadic patients of ANSD and congenital SNHL [20]–[22].

The *PJVK* gene is responsible for 4 Iranian families with DFNB59 (MIM#610220), and all of them were diagnosed with ANSD [11]. Homozygous *PJVK-*mutated knock-in mice showed mimic phenotype of patients with *PJVK* mutations, such as abnormal ABR and preserved function of IHCs and OHCs. The pejvakin, encoded by *PJVK,* was detected in the cell bodies of neurons in the auditory pathway and is believed to be essential in neural activity [11].

Cochlear implant could be the last resort for patients with ANSD [23] because hearing aids or medicines are not beneficial to these patients. Although the outcome in children afflicted with ANSD is not as good as those with SNHL [24], improvement of speech perception following cochlear implants has been reported in some patients [25]. For ANSD patients, the result of cochlear implant may depend on the location of the lesion. Cochlear implants provide supraphysiologic electrical stimulation of the auditory nerve and may improve the synchronicity of the neural activity. Therefore, cochlear implants may be beneficial for pre-synaptic ANSD, contrary to post-synaptic ANSD [23]. Otoferlin and pejvakin work in functionally separate cells. Hence, we infer that gene screening may be an advantageous method to identify the subtypes of non-syndromic ANSD and could be a guide for cochlear implants. Cochlear implantation actually showed good outcomes in subjects with *OTOF* mutations [26], [27].

Sporadic hearing loss could be the result of non-genetic factors as well as recessive gene mutations. *GJB2* gene mutations can cause congenital and postlingual hearing loss, with pure-tone threshold ranging from moderate to profound [28]. Until recently, almost all mutations responsible for ANSD are detected in congenital patients. In reality, there is inadequate information regarding the genetic data in postlingual ANSD patients, especially those whose hearing disorder is manifested in their second or third decade. The aim of the present study was to evaluate the variations in *OTOF*, *PJVK*, and *GJB2 genes*, as well as in mitochondrial *12S rRNA*, in sporadic postlingual ANSD patients living in northwest China.

## Methods

### Patient Recruitment and Clinical Evaluations

We enrolled 76 unrelated patients diagnosed with non-syndromic ANSD from January 1, 2009 to May 20, 2011. All the patients were of Chinese Han ethnicity living in northwest China. The case group comprised 45 females and 31 males, with onset age of hearing loss varying from 5 to 32 years (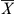 = 16.2±4.6). All the patients denied any history of ototoxic drug or noise exposure. Syndromic disorders were previously ruled out in all cases by inquiry and physical examinations. The control group consisted of 105 matched normal-hearing subjects.

The diagnostic criteria of ANSD was defined briefly as follows [4], [5]: (1) complaints of hearing loss, with difficulty in word discrimination; (2) normal or partially normal distortion product otoacoustic emissions and tympanometry, but abnormal ABR and stapedial reflexes; (3) pure-tone audiometries show SNHL; and (4) negative in computerized tomography scanning of the temporal bone and/or magnetic resonance hydrography of the inner ear.

The present study was approved by the Institutional Review Board of the Ethics Committee of China People's Liberation Army Xijing Hospital, the Fourth Military Medical University. Informed consent was signed by the participants and/or their parents prior to obtaining their blood samples for the genetic study.

### Genes sequencing

Genomic DNA samples of the participants were extracted from peripheral venous blood by standard procedures of the Bioteke kit (DP2102, Bioteke Corp.). We designed 41 pairs and 6 pairs of primers to amplify DNA fragments containing all exons in the coding regions of gene *OTOF* and *PJVK*, respectively (**Table S1 and** **Table S2** in supplementary document). All the primers were designed using Primer 3.0 online software and synthesized by Shenggong DNA Technologies. Polymerase chain reactions (PCR) were performed in a total volume of 50 μL by 2 × Power Taq PCR MasterMix (PR1700, Bioteke Corp.) with 100 ng of genomic DNA in MyCycler thermocyclers (Bio-rad Corp.). PCR products were finally sequenced by ABI 3730Xl DNA Analyzer. The data were subsequently compared with the wild-type *OTOF* (NG_009937.1, NM_194248.2) and *PJVK* (NG_0012186.1, NM_001042702.3) sequences, and a panel of 105 controls verified it. Variations corresponding with the coding region were numbered as in the otoferlin isoform a (NP_919224.1) and pejvakin (NP_001036167.1). Meanwhile, the patients were also subjected to mutation screening for genes commonly associated with NSHL and some patients of ANSD, namely, the *GJB2* and the mitochondrial *12S rRNA* genes.

## Results

### Identification of *OTOF* Gene Variants

**Figure 1:**
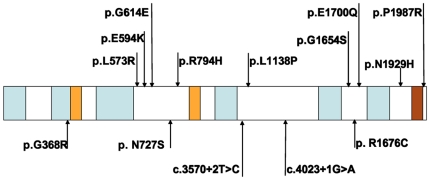
Novel pathogenic *OTOF* mutations in this work and the relation to the functional domains [**42**]. Blue: 6 C2 domains. Orange: FerI and FerB domains. Brown: transmembrane domain. Variations of this work are noted under the schematic diagram, and other missense mutations outside the function domains are shown above it.

**Figure 2:**
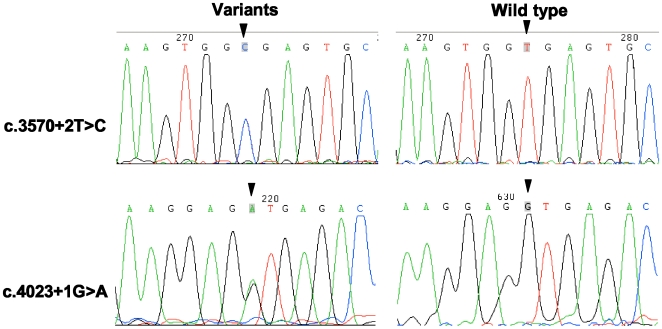
Sequencing chromatograms of novel splice site variants identified in the present work with wild type control. The variants include c.3570+2T>C and c.4023+1G>A. Corresponding variant locations are arrowed.

**Figure 3:**
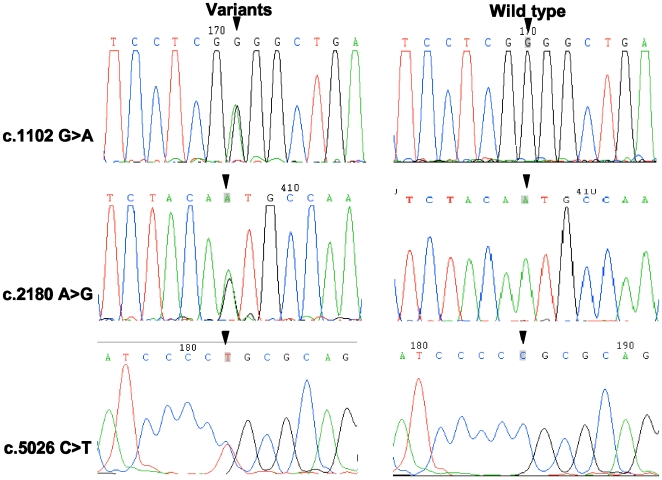
Sequencing chromatograms of novel missense mutations identified in the present work with wild type control. The variants include c.1102G>A, c.2180A>G and c.5026C>T. Corresponding variant locations are arrowed.

Among 70 ANSD patients, 7 novel variants were found in the *OTOF* gene, including 2 splice site mutations, 3 missense mutations, and 2 silent variants (**Table 1** and **Figure 1**, and **Table S3** in supplementary document). The splice site mutations were as follows: (1) c.3570+2T>C, a T to C transition at the donor site of intron 28; and (2) c.4023+1G>A, a G to A transition at the donor site of intron 32 (**Figure 2**). The missense mutations were as follows: (1) c.1102G>A (p.G368R), a transition from G to A in exon 12 caused a substitution from a nonpolar neutral amino acid glycine to a polar positive amino acid arginine; (2) c.2180A>G (p.N727S), a transition from A to G in exon 18 led to a substitution from asparagine to serine; and (3) c.5026C>T (p.R1676C), a transition from C to T in exon 40 resulted in a substitution from a polar positive amino acid arginine to a nonpolar neutral amino acid cysteine (**Figure 3**). Silent variations include c.3615G>A and c.3684T>A in isoform d mRNA (NM_194323.2, **Table S3** in supplementary document). In addition, we identified a missense mutation c.1194T>A (p.D398E) in one patient, which had been reported as pathogenic mutation by other researchers [21]. All these variations were heterozygously carried by different patients.

**Table 1:** Probable-pathogenic variants of *OTOF* identified in 76 patients.

Location	Nucleotide change	Codon change	Occurrence in the present work	Occurrence in control	References
Exon 12	c.1102G>A	p.G368R	1/76	0/105	Present work
Exon 12	c.1194T>A	p.D398E	1/76	0/105	[16]
Exon 18	c.2180A>G	p.N727S	1/76	0/105	Present work
Intron 28	c.3570+2T>C	Splice site	1/76	0/105	Present work
Intron 32	c.4023+1G>A	Splice site	1/76	0/105	Present work
Exon 40	C.5026C>T	p.R1676C	1/76	0/105	Present work

**Figure 4:**
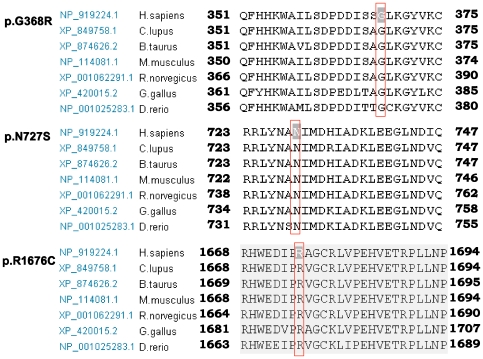
Evolutionary conservation of the residue altered in novel *OTOF* mutations. The ClustalW alignment result of the long isoform of otoferlin among different organisms, including human, dog, cattle, mouse, rat, bird and fish. The locations of mutated amino acids are boxed and highlight in inversed color.

We assumed that the splice site mutations and the missense variants c.1102G > A (p.G368R) were possibly pathogenic because (1) splice site mutations yielded an incorrect splicing of mRNA and the missense mutation was located in the functional C2B regions, which are highly conserved among vertebrate species (**Figure 4**), and (2) none of these mutations were observed in 105 unrelated controls (**Table 1**). The other novel variations, c.2180A>G (p.N727S) and c.5026C>T (p.R1676C), were also observed to be highly conserved from zebrafish to humans, and were absent in the control (**Table 1** and **Figure 4**). However, whether they are harmless variants or pathogenic mutations is difficult to elucidate.

Moreover, 15 variants encoding polymorphic changes were identified, including 4 missense variants, namely, c.157G>A (p.A53T), c.158C>T (p.A53V), c.244C>T (p.R82C) and c.4936C>T (p.P1646S); all of which have been reported previously [21] (**Table S3** in supplementary document). We identified 28 variants in the flanking regions of exons, including 7 novel variants, namely, g.336G>A, g.21153G>C, g.21254T>G, g.77602G>C, g.81074G>A, g.87599G>A, and g.100441G>A.

### Identification of *PJVK* Gene Variants

**Figure 5:**
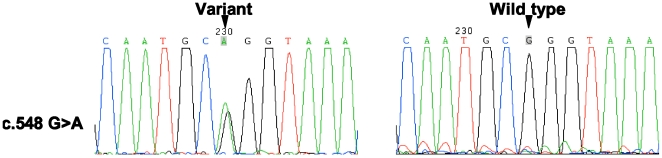
Sequencing chromatograms of novel pathogenic variant (c.548G>A) in this work with control. Arrow: the variant location.

We found 3 novel variants in the exons of the *PJVK* gene, including 1 missense mutation and 2 silent mutations, which are as follows: (1) c.548G>A (p.R183Q), a transition from G to A in exon 4 caused a substitution from a basic amino acid arginine to a neutral amino acid glutamine (**Figure 5**); and (2) c.921G>C and c.*2 A>C were silent variants in exon 7 (**Table 2**). All these variations were heterozygously carried by different patients. We also identified a polymorphic variant c.874G>A (p.G292R) in exon 7 [29] and two variants, g.1611G>A and g.5337C>T, in the introns of *PJVK*.

**Table 2:** Genetic variants of *PJVK* identified in 76 patients.

Location	Nucleotide change	Codon change	Occurrence in this work	Occurrence in control	References
Exon 4	c.548 G>A	p.R183Q	1/70	0/105	This work
Exon 7	c.874 G>A	p.G292R	19/70	21/105	[41]
Exon 7	c.921G>C	p.G307G	1/70	0/105	This work
3′ UTR	c.*A>C		1/70	1/105	This work

**Figure 6:**
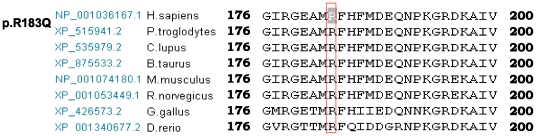
Evolutionary conservation of the residue altered in the *PJVK* gene. The ClustalW of pejvakin are shown among different organisms, including human, Chimpanzee, dog, cattle, mouse, rat, bird and fish. The locations of mutated amino acids are boxed and highlight in inversed color.

We assumed that the missense mutation c.548G>A (p.R183Q) was possibly pathogenic because: (1) the region is highly conserved among vertebrate species (**Figure 6**); (2) the mutation was not observed in 105 unrelated controls (**Table 2**); and (3) previous study has identified c.547C>T (p.R183W) as a pathogenic mutation, which emphasized the importance of the conservation of this region [11].

### Identification of *GJB2* gene and mitochondrial *12S rRNA* Variants

We did not detect known pathogenic variations or novel mutation in the gene *GJB2* and mitochondrial *12S rRNA*. Some variants encoding polymorphic changes were detected, including c.79G>A, c.109G>A and c.341A>G in gene *GJB2* and c.663A>G, c.709G>A, c.750A>G, c.1107T>C and c.1438A>G in the mitochondrial *12S rRNA* gene.

## Discussion

ANSD is closely related to the *OTOF* gene, which contains 47 coding exons and spans approximately 90 kb, encoding protein otoferlin [12]. Otoferlin is a fer-1-like protein, containing a transmembrane domain and 6 C2 domains [30]. In situ hybridization in neonatal mice cochlea, otoferlin labeling was dominantly seen in the IHCs, but faintly in the OHCs, spiral ganglion cells, as well as in the neuroepithelia hair cells of the utricle, saccule, and semicircular canals [31]. Roux et al. [19] observed that otoferlin was localized mainly in the ribbon-associated synaptic vesicles. Data showed that *otof*erlin is crucial in IHCs exocytosis and membrane fusion in a Ca^2+^-sensitive manner during hearing message transmission at the ribbon synapse [19].

The *OTOF* gene was first cloned and identified in a Lebanese family with DFNB9 non-syndromic hearing loss, which was not originally diagnosed as ANSD [15]. Varga et al. [12] studied four families with ANSD by linkage and mutation analyses and found that the *OTOF* gene is responsible for the hearing loss in three of the families. Furthermore, Migliosi et al. [32] identified a frequent *OTOF* mutant, c.2485C>T (p.Q829X), in Spanish patients with NSHL. An international multicenter study confirmed the prevalence and spectrum of *OTOF* mutation in Spanish families or subjects of Spanish ancestry with NSHL [17]. The mutation of p.Q829X in the *OTOF* gene is responsible for 2.3%–5.1% NSHL patients [17], most of who are diagnosed with ANSD. However, the mutation spectrum of the *OTO*F gene in the Pakistani and Brazilian populations are evidently different from those reported in whites [16], [33]. Recently, an *OTOF* variant study also showed a different mutation spectrum in ANSD patients in Taiwan and east China [21], [22].

In the present study, we identified 2 novel splice site mutations and 3 novel missense mutations in the *OTOF* gene within 76 sporadic postlingual ANSD patients. However, we did not detect other pathologic allelic variants reported previously, not even the most frequently reported mutations, p.Q829X in Spanish patients and p.E1700Q in the Taiwanese subjects, who are closer genetically to our patients [34]. We detected a similar mutation as reported in a study about ANSD patients east China [21]. We assumed that the novel splice site mutations and the missense variant c.1102G>A (p.G368R) are possibly pathogenic because they are functionally important and highly conserved among species. The other 2 novel variations were located outside the functional domains. Although the amino acids are also demonstrated to be highly conserved from the zebrafish to humans, and absent in the control, and a batch of pathogenic missense mutations were located outside the functional domains, whether these variations are harmless variants or pathogenic mutations are difficult to establish because we were unable to find other pathogenic mutations.

*PJVK* gene, encoding protein pejvakin, consists of 7 exons spanning 9.8 kb of genomic sequence. Homozygous *PJVK*-mutated knock-in mice, qualified with ANSD, exhibited abnormal ABR indicative of neuronal dysfunction along the auditory pathway and normal otoacoustic emissions that indicated well preserved hair cells [11]. Pejvakin was detected in the cell bodies of neurons of the afferent auditory pathway. Delmaghani et al. [11] concluded that pejvakin is essential in the auditory pathway neuronal activity. Apparently, the *PJVK* gene is important in ANSD patients, although its mutations are also related to SNHL [29], [35]. Furthermore, pejvakin- mutated mice showed SNHL with preserved IHCs and OHCs [11], [36].

Delmaghani et al. [11] detected the R183W mutation in a recessive ANSD family and proved that R183W mutation is pathogenic in the knock-in mouse model. At the same position, we detected a missense mutation c.548G>A (p.R183Q), which altered arginine to glutamine. We believed that the R183Q variation is another pathogenic mutation in the *PJVK* gene. We did not detect other pathogenic mutations in our patients. Collin et al. [29] analyzed the carrying rate of *PJVK* mutations in 151 patients, and concluded that the *PJVK* gene mutations do not primarily cause NSHL. Nevertheless, considering the specificity of cells where the *PJVK* gene functioned [11], the mutation spectra in ANSD patients should be monitored continuously.

*GJB2* gene and mitochondrial*12S rRNA* gene are two of the most prevalent pathogenic genes responsible for NSHL [37], and mutations in both genes had been identified in sporadic AN patients [13], [14]. However, we did not detect pathogenic mutations in the *GJB2* gene or the *12S rRNA* gene in our patients. Additional ANSD patients should be enrolled to study the relationship between these two genes and ANSD.

Clinically, two single heterozygous genes, *GJB2* and *GJB6*, have been found responsible for NSHL patients [38]. In patients with enlarged vestibular aqueduct syndrome, both single heterozygous genes *SLC26A4* (MIM*605646) and *KCNJ10* (MIM*602208) induced the hearing loss in these patients [39]. In a previous study, a single heterozygous variant in *OTOF* gene is linked as pathogenic [12]. In the present study, all patients carried single heterozygous pathogenic variants of gene *OTOF* or *PJVK.* We supposed the possibility of other genes participating in the ANSD pathogenesis cooperation with *OTOF* and *PJVK* or that the disorder resulted from a combination of genetic and environmental conditions. Candidate genes for exploration could be the genes encoding ion channel proteins or genes participating in the mitochondria [40]. On the other hand, copy number variations could be another possibility in patients with or without heterozygous variants because the deletion of exons or the entire gene in one or both allele may be missed by amplification and direct sequencing. Our future work would focus on the detection of copy number variations in patients with single heterozygous variants. Furthermore, most of our patients live in the mountain and rural areas; thus, environmental etiology should also be considered.

Santarelli et al. [33] noted that ANSD patients with *OTOF* gene mutations exhibit some variations from other ANSD patients, which could predict the results of cochlear implantation [26], [27]. However, none of our patients with *OTOF* mutations underwent operation for cochlear implantation, considering the pressure of economy and the risk of inefficiency. Two ANSD patients without variants in either of the genes had cochlear implant. A 5-year-old girl with profound hearing loss significantly improved, in contrast to a 15-year old boy with moderate hearing who showed no improvement in words understanding. The difference in their results still needs to be explored in further studies.

In conclusion, we identified 3 novel possible pathogenic variants and 1 known probable pathogenic mutation of the *OTOF* gene in 76 Chinese ANSD patients. We identified 1 novel possible pathogenic variant of the *PJVK* gene in the same group. The special mutation spectra of these 2 genes in Chinese subjects could be the result of across-race diversity and could be beneficial in understanding the worldwide occurrence of ANSD.

## Supporting Information

Table S1 **PCR primers for OTOF gene screening.**
(DOC)

Table S2 **PCR primers for PJVK gene screening.**
(DOC)

Table S3 **Non-pathogenic variants of OTOF gene identified in this study.**
(DOC)

## References

[1] Berlin CI, Hood L, Morlet T, Rose K, Brashears S (2003). Auditory neuropathy/dys-synchrony: diagnosis and management.. Ment Retard Dev Disabil Res Rev.

[2] Starr A, Picton TW, Sininger Y, Hood LJ, Berlin CI (1996). Auditory neuropathy.. Brain.

[3] Manchaiah VK, Zhao F, Danesh AA, Duprey R (2010). The genetic basis of auditory neuropathy spectrum disorder (ANSD)..

[4] Starr A, Sininger YS, Pratt H (2000). The varieties of auditory neuropathy.. J Basic Clin Physiol Pharmacol.

[5] Wang J, Gao L, Xue F, Meng M, Zha D (2002). [Auditory neuropathy].. Lin Chuang Er Bi Yan Hou Ke Za Zhi.

[6] Tang TP, McPherson B, Yuen KC, Wong LL, Lee JS (2004). Auditory neuropathy/auditory dys-synchrony in school children with hearing loss: frequency of occurrence.. Int J Pediatr Otorhinolaryngol.

[7] Rance G, Beer DE, Cone–Wesson B, Shepherd RK, Dowell RC (1999). Clinical findings for a group of infants and young children with auditory neuropathy.. Ear Hear.

[8] Wang J, Shi L, Xue F, Sun W, Gao L (2007). Differential Audiological Assessment of Patients with Auditory Neuropathy.. Journal of Audiology and Speech Pathology.

[9] Kirkim G, Serbetcioglu B, Erdag TK, Ceryan K (2008). The frequency of auditory neuropathy detected by universal newborn hearing screening program.. Int J Pediatr Otorhinolaryngol.

[10] Robertson CM, Howarth TM, Bork DL, Dinu IA (2009). Permanent bilateral sensory and neural hearing loss of children after neonatal intensive care because of extreme prematurity: a thirty-year study.. Pediatrics.

[11] Delmaghani S, del CF, Michel V, Leibovici M, Aghaie A (2006). Mutations in the gene encoding pejvakin, a newly identified protein of the afferent auditory pathway, cause DFNB59 auditory neuropathy.. Nat Genet.

[12] Varga R, Kelley PM, Keats BJ, Starr A, Leal SM (2003). Non-syndromic recessive auditory neuropathy is the result of mutations in the otoferlin (OTOF) gene.. J Med Genet.

[13] Cheng X, Li L, Brashears S, Morlet T, Ng SS (2005). Connexin 26 variants and auditory neuropathy/dys-synchrony among children in schools for the deaf.. Am J Med Genet A.

[14] Wang Q, Li R, Zhao H, Peters JL, Liu Q (2005). Clinical and molecular characterization of a Chinese patient with auditory neuropathy associated with mitochondrial 12S rRNA T1095C mutation.. Am J Med Genet A.

[15] Yasunaga S, Grati M, Cohen–Salmon M, El–Amraoui A, Mustapha M (1999). A mutation in OTOF, encoding otoferlin, a FER-1-like protein, causes DFNB9, a nonsyndromic form of deafness.. Nat Genet.

[16] Romanos J, Kimura L, Favero ML, Izarra FA, de Mello AM (2009). Novel OTOF mutations in Brazilian patients with auditory neuropathy.. J Hum Genet.

[17] Rodriguez–Ballesteros M, Reynoso R, Olarte M, Villamar M, Morera C (2008). A multicenter study on the prevalence and spectrum of mutations in the otoferlin gene (OTOF) in subjects with nonsyndromic hearing impairment and auditory neuropathy.. Hum Mutat.

[18] Zanazzi G, Matthews G (2009). The molecular architecture of ribbon presynaptic terminals.. Mol Neurobiol.

[19] Roux I, Safieddine S, Nouvian R, Grati M, Simmler MC (2006). Otoferlin, defective in a human deafness form, is essential for exocytosis at the auditory ribbon synapse.. Cell.

[20] Zadro C, Ciorba A, Fabris A, Morgutti M, Trevisi P (2010). Five new OTOF gene mutations and auditory neuropathy.. Int J Pediatr Otorhinolaryngol.

[21] Wang DY, Wang YC, Weil D, Zhao YL, Rao SQ (2010). Screening mutations of OTOF gene in Chinese patients with auditory neuropathy, including a familial case of temperature-sensitive auditory neuropathy.. BMC Med Genet.

[22] Chiu YH, Wu CC, Lu YC, Chen PJ, Lee WY (2010). Mutations in the OTOF gene in Taiwanese patients with auditory neuropathy.. Audiol Neurootol.

[23] Gibson WP, Graham JM (2008). Editorial: ‘auditory neuropathy’ and cochlear implantation - myths and facts.. Cochlear Implants Int.

[24] Miyamoto RT, Kirk KI, Renshaw J, Hussain D (1999). Cochlear implantation in auditory neuropathy.. Laryngoscope.

[25] Rance G, Barker EJ (2008). Speech perception in children with auditory neuropathy/dyssynchrony managed with either hearing AIDS or cochlear implants.. Otol Neurotol.

[26] Rodriguez–Ballesteros M, del CF, Martin Y, Moreno–Pelayo MA, Morera C (2003). Auditory neuropathy in patients carrying mutations in the otoferlin gene (OTOF).. Hum Mutat.

[27] Rouillon I, Marcolla A, Roux I, Marlin S, Feldmann D (2006). Results of cochlear implantation in two children with mutations in the OTOF gene.. Int J Pediatr Otorhinolaryngol.

[28] Pollak A, Skórka A, Mueller–Malesińska M, Kostrzewa G, Kisiel B (2007). M34T and V37I mutations in GJB2 associated hearing impairment: evidence for pathogenicity and reduced penetrance.. Am J Med Genet A.

[29] Collin RW, Kalay E, Oostrik J, Caylan R, Wollnik B (2007). Involvement of DFNB59 mutations in autosomal recessive nonsyndromic hearing impairment.. Hum Mutat.

[30] Yasunaga S, Grati M, Chardenoux S, Smith TN, Friedman TB (2000). OTOF encodes multiple long and short isoforms: genetic evidence that the long ones underlie recessive deafness DFNB9.. Am J Hum Genet.

[31] Beurg M, Safieddine S, Roux I, Bouleau Y, Petit C (2008). Calcium- and otoferlin-dependent exocytosis by immature outer hair cells.. J Neurosci.

[32] Migliosi V, Modamio–Høybjør S, Moreno–Pelayo MA, Rodríguez–Ballesteros M, Villamar M (2002). Q829X, a novel mutation in the gene encoding otoferlin (OTOF), is frequently found in Spanish patients with prelingual non-syndromic hearing loss.. J Med Genet.

[33] Choi BY, Ahmed ZM, Riazuddin S, Bhinder MA, Shahzad M (2009). Identities and frequencies of mutations of the otoferlin gene (OTOF) causing DFNB9 deafness in Pakistan.. Clin Genet.

[34] McClellan J, King MC (2010). Genetic heterogeneity in human disease.. Cell.

[35] Ebermann I, Walger M, Scholl HP, Charbel IP, Luke C (2007). Truncating mutation of the DFNB59 gene causes cochlear hearing impairment and central vestibular dysfunction.. Hum Mutat.

[36] Schwander M, Sczaniecka A, Grillet N, Bailey JS, Avenarius M (2007). A forward genetics screen in mice identifies recessive deafness traits and reveals that pejvakin is essential for outer hair cell function.. J Neurosci.

[37] Hilgert N, Smith RJ, Van Camp G (2009). Forty-six genes causing nonsyndromic hearing impairment: which ones should be analyzed in DNA diagnostics?. Mutat Res.

[38] Belintani PV, Maria GBE, Lucia SE, Victor MJ (2004). Prevalence of the GJB2 mutations and the del(GJB6-D13S1830) mutation in Brazilian patients with deafness.. Hear Res.

[39] Yang T, Gurrola JN, Wu H, Chiu SM, Wangemann P (2009). Mutations of KCNJ10 together with mutations of SLC26A4 cause digenic nonsyndromic hearing loss associated with enlarged vestibular aqueduct syndrome.. Am J Hum Genet.

[40] Cacace AT, Pinheiro JM (2011). The Mitochondrial Connection in Auditory Neuropathy.. Audiol Neurootol.

[41] Hashemzadeh, M C (2007). Novel mutations in the pejvakin gene are associated with autosomal recessive non-syndromic hearing loss in Iranian families.. Clin Genet.

[42] Jiménez JL, Bashir R (2007). In silico functional and structural characterisation of ferlin proteins by mapping disease-causing mutations and evolutionary information onto three-dimensional models of their C2 domains.. J Neurol Sci.

