# Supplementary material for: Variants of OTOF and PJVK Genes in Chinese Patients with Auditory Neuropathy Spectrum Disorder
Source: PLoS One. 2011 Sep 15;6(9):e24000. doi: 10.1371/journal.pone.0024000 (PMC3174136; doi:10.1371/journal.pone.0024000)
Supplement: Table S1 — PCR primers for OTOF gene screening. (DOC) [file pone.0024000.s001.doc]

**Table S1. PCR primers for OTOF gene screening.**

| Exon | Forward primer | Reverse primer | Product size (bp) |
| --- | --- | --- | --- |
| 1 | 5’- GGAGGAGGCAGCGGCAGAG-3’ | 5’-ATGTTGGCAAGCACAAGGAGAC-3’ | 400 |
| 2 | 5’-AAATGCTGTTAGGACGACTC-3’ | 5´-CTCCACAGGGAATAACAGG-3´ | 435 |
| 3 | 5´-AGGTGGTTGTTGGAACTAAATG-3´ | 5´-GCCCAAGGAGAAGAGCAGG-3´ | 313 |
| 4 | 5´-CCTCCCCAAGCAGTCACAG-3´ | 5´-CATTCCCCAGACCACCCCA-3´ | 266 |
| 5 | 5´-CAAGTCAGCCTTTCAGAGC-3´ | 5´-GTCCTGCCTGTTACCTCC-3´ | 363 |
| 6 | 5´-TGGCAAAGGCGGAAGTGGAG-3´ | 5´-CCTAGAGGGCCACGCATCACTG-3´ | 420 |
| 7 | 5´-TAAGCGGTAGCAACTAAGTAAAC-3´ | 5´-GAGCCCTGATTCTTCCCTAC-3´ | 330 |
| 8 | 5´-CTTGGCAGTGCTTGAGTG-3´ | 5´-GATCCATGCCTCAGTATAGTG-3´ | 264 |
| 9 | 5´-AGAGGCAGTGGTCAGAATGG-3´ | 5´-GTGGCTCTGTTTGTCAGTGTC-3´ | 426 |
| 10 | 5´-GCTGCTCCTCTGAACTTTGG-3´ | 5´-CCACTGTTTACGGGCGAAT-3´ | 393 |
| 11 | 5´-CTCCCACTTCACCACAAAGCTC-3´ | 5´-TGGTCCTTGCCAGCCTTTTC-3´ | 269 |
| 12 | 5´-ACTCTAGGGACCAAGACAGC-3´ | 5´-CTGGCAGAAAGGATGTCAAAG-3´ | 467 |
| 13 | 5´-TAGCCTTGCTCCCTCAG-3´ | 5´-TTTCCAGCCTTGTCTTACC-3´ | 396 |
| 14 | 5´-ACATGCCACGCCCTCACCT-3´ | 5´-GAGAGGGCATCCACATATTC-3´ | 301 |
| 15 | 5´-GCAGGGAAGAAATAGACCCAAGA-3´ | 5´-GAAGTTCTCAGCTCAGCACCCA-3´ | 538 |
| 16 | 5´-CGGCTGTCCCTGTGGTGTCT-3´ | 5´-GCCTGCCTGTGCTCTGTCTC-3´ | 417 |
| 17-18 | 5´-TTCCCAAACCCAAGGCAGCAC-3´ | 5´-TGGCTCCTGTCCTTGTCTGTG-3´ | 636 |
| 19 | 5´-CAGCATTCTGGAGTGACGTC-3´ | 5´-GGGCTCTGTAGATTCTTCCTC-3´ | 555 |
| 20-21 | 5´-GCTGGGCAGATGAGGAAG-3´ | 5´-CACGCTTGTTGTTGCTCATC-3´ | 572 |
| 22 | 5´-AAGCTGAGGCTGTGCCAGAA-3´ | 5´-TTCCCATTCTTGGCTCTTCTC-3´ | 449 |
| 23-24 | 5´-CCCATCCTCCTGCCTCCAC-3´ | 5´-AGCCCGTAGCCTTTCCAGTG-3´ | 731 |
| 25 | 5´-TTTCTGGAAGAATCAAGACAC-3´ | 5´-CAGGAGGAGCTAGATGTCA-3´ | 421 |
| 26 | 5´-CTGTAAAATGGGGTGATCACA-3´ | 5´-CCCCAGGACCTAACGC-3´ | 478 |
| 27 | 5´-GCCTCTCTCCTACCCATCCT-3´ | 5´-CTGCTGGCTCCTGGTGAT-3´ | 353 |
| 28-29 | 5´-CCGTGAGTTCTGCCCAGGCC-3´ | 5´- CTTGGACTGGGCGGAGAC -3´ | 633 |
| 30 | 5´-GCTCCCACCCTTTGAAATCT-3´ | 5´-GATGTGTCACACGAAGTTGCATGTT -3´ | 238 |
| 31 | 5´- AATTTCCATCTCGGCTCCTC -3´ | 5´- CCTTTGGCCTGACATCATTG -3´ | 182 |
| 32 | 5´- GCTGACAGATGGCGGAATG -3´ | 5´- CGTGGGAAAGAAGCTGGA -3´ | 270 |
| 33 | 5´- GAGCACATGGTGGACTTGAA -3´ | 5´- GGGAAAAGAGAAGCAGGTGA -3´ | 279 |
| 34-35 | 5´- CGGGCATGCTAGGGAGTAT -3´ | 5´- GCTGAGTCATGGGAGAGTCC -3´ | 664 |
| 36 | 5´- CTCCTGGTGCTGTTAGCTAT -3´ | 5´- GATGAGGAGACTTGCAAGGAG -3´ | 237 |
| 37 | 5´- CCCCTGCCCTTCACTGTCATA -3´ | 5´- GAAGGAGGCCTCGATGTCAA -3´ | 341 |
| 38 | 5´- CCACCCTAGCCAATCCTTA -3´ | 5´- AATCACCAGGATCTGAATCTC -3´ | 279 |
| 39 | 5´- GAGGAATCAGGCTCACTTGC-3´ | 5´- CCAGGTTTAGGCTGAGGACA-3´ | 387 |
| 40 | 5´- TAGACAGGTGATGGCATAGAG-3´ | 5´- GCCTGGCTTCTACCTTTAC-3´ | 318 |
| 41 | 5´- TTAGGAGGGAGAGGAGAGC-3´ | 5´- AAGGCCATCTGGACCTGAG-3´ | 248 |
| 42 | 5´- GCCTTCTCACTCATGCCTCT-3´ | 5´- GAACAGGTAGCGCCAGTTG-3´ | 498 |
| 43 | 5´- GAGGAGGCAGAGGGAAGG-3´ | 5´- GATGGACTGGAAGCAATGAC-3´ | 423 |
| 44-45 | 5´- TGCCTAGCAAGCCCACGAG-3´ | 5´- GTCTGGGGATCGTCTCCTT-3´ | 625 |
| 46 | 5´-CGGAGAGAAACTATGCTC-3´ | 5´- TTGTGGATGTGTGCGTGTATA-3´ | 475 |
| 47 | 5´-CCCTGGCTCCAGCATTCAC-3´ | 5´- GGAGATGGGAAAGAGTCCAAG-3´ | 373 |
