# Supplementary material for: Variants of OTOF and PJVK Genes in Chinese Patients with Auditory Neuropathy Spectrum Disorder
Source: PLoS One. 2011 Sep 15;6(9):e24000. doi: 10.1371/journal.pone.0024000 (PMC3174136; doi:10.1371/journal.pone.0024000)
Supplement: Table S2 — PCR primers for PJVK gene screening. (DOC) [file pone.0024000.s002.doc]

**Table S2. PCR primers for PJVK gene screening[[1]](#endnote-2).**

| Exon | Forward primer | reverse primer | Product size (bp) |
| --- | --- | --- | --- |
| 2 | 5’-TGTTTGCTGCTGCTACC-3’ | 5’-TTGGCATTGTTAATCTTAATA-3’ | 352 |
| 3 | 5’-ACATTTGGGTATTTGAGTCTT-3’ | 5´-GCCAAGTGATTTTTGATATCT-3´ | 464 |
| 4 | 5´-ATTGCCTTGATTTACTATTAG-3´ | 5´-CACAAAATACACAAGCCT-3´ | 338 |
| 5 | 5´-CTGTTGGACCAATTGGATCTC-3´ | 5´-TCATGCAGACCCTTAACTCAC-3´ | 294 |
| 6 | 5´-CCAAAAGTATGTAAAAGTCAA-3´ | 5´-TCCTTAACAGATGAGATTACT-3´ | 301 |
| 7 | 5´-AGCCAAATTATTTCATGACTA-3´ | 5´-ACTGCACCTAAAACACCAAC-3´ | 424 |

1. Exon1 is out of coding region [↑](#endnote-ref-2)
