# Supplementary material for: Variants of OTOF and PJVK Genes in Chinese Patients with Auditory Neuropathy Spectrum Disorder
Source: PLoS One. 2011 Sep 15;6(9):e24000. doi: 10.1371/journal.pone.0024000 (PMC3174136; doi:10.1371/journal.pone.0024000)
Supplement: Table S3 — Non-pathogenic variants of OTOF gene identified in this study. (DOC) [file pone.0024000.s003.doc]

**Table S3. Non-pathogenic variants of OTOF gene identified in this study.**

| Location | Nucleotide change | Codon change | Occurence | References |
| --- | --- | --- | --- | --- |
| Exon 2 | c.129C>T | R43W | 13 | [1] |
| Exon 3 | c.157G>A | A53T | 3 | [1] |
| Exon 3 | c.158C>T | A53V | 25  1 | Rs1879761[[1]](#footnote-2) |
| Exon 4 | c.244C>T | R82C | 10  1 | Rs13031859 |
| Exon 5 | c.372A>G | 124T | 35  19 | Rs11687696 |
| Exon 22 | c.2580C>G | 860V | 8  22 | Rs2272069 |
| Exon 22 | c.2613C>T | 871L | 7  1 | Rs2272068 |
| Exon 23 | c.2703G>A | 901S | 3  1 | Rs4997760 |
| Exon 23 | c.2736G>C | 912L | 24 | Rs4335905 |
| Exon 38 | c.4677G>A | 1559V | 7  1 | Rs2272071 |
| Exon 38 | c.4767C>T | 1589R | 6 | Rs80356578 |
| Exon 39 | c.4936C>T | P1646S | 1 | Rs17005371 |
| Exon 43 | c.5391C>T | 1797F | 1 | Rs61747275 |
| Exon 43 | c.5418C>T | 1806I | 6 | [1] |
| Exon 44 | c.5655C>T | 1885R | 1 | Rs45442103 |
| Exon 47d[[2]](#footnote-3) | c.3651G>A | 1217A | 1 | This work |
| Exon 47d^3^ | c.3684T>A | 1227L | 1 | This work |

References:

[1]. Wang, D.Y., et al., Screening mutations of OTOF gene in Chinese patients with auditory neuropathy, including a familial case of temperature-sensitive auditory neuropathy. BMC Med Genet, 2010. 11: p. 79.

1. registered variations in dbSNP database [↑](#footnote-ref-2)
2. otoferlin isoform d mRNA(NM_194323.2)，lies in the exon47 of otoferlin isoform a [↑](#footnote-ref-3)
